# Pharmacological Profile of Xanthohumol, a Prenylated Flavonoid from Hops (*Humulus lupulus*)

**DOI:** 10.3390/molecules20010754

**Published:** 2015-01-07

**Authors:** Ming Liu, Poul Erik Hansen, Genzhu Wang, Lin Qiu, Jianjun Dong, Hua Yin, Zhonghua Qian, Mei Yang, Jinlai Miao

**Affiliations:** 1 Key Laboratory of Marine Drugs, Ministry of Education, School of Medicine and Pharmacy, Ocean University of China, Qingdao 266003, China; E-Mail: wanggenzhu_890813@163.com; 2 Department of Science, Systems and Models, Roskilde University, P.O. Box 260, DK-4000 Roskilde, Denmark; E-Mail: poulerik@ruc.dk; 3 Institute for Nutritional Sciences, Shanghai Institute for Biological Sciences, Chinese Academy of Sciences, Shanghai 200031, China; E-Mail: qiulin@sibs.ac.cn; 4 State Key Laboratory of Biological Fermentation Engineering of Beer, Qingdao 266061, China; E-Mails: 15133634098@163.com (J.D.); yinhua@tsingtao.com.cn (H.Y.); qianzh@tsingtao.com.cn (Z.Q.); yangmei@tsingtao.com.cn (M.Y.).; 5 Key Laboratory of Marine Bioactive Substance, The First Institute of Oceanography, State Oceanic Administration, Qingdao 266061, China

**Keywords:** biological effects, pharmacokinetics, food additives, beer, metabolites

## Abstract

The female inflorescences of hops (*Humulus lupulus* L.), a well-known bittering agent used in the brewing industry, have long been used in traditional medicines. Xanthohumol (XN) is one of the bioactive substances contributing to its medical applications. Among foodstuffs XN is found primarily in beer and its natural occurrence is surveyed. In recent years, XN has received much attention for its biological effects. The present review describes the pharmacological aspects of XN and summarizes the most interesting findings obtained in the preclinical research related to this compound, including the pharmacological activity, the pharmacokinetics, and the safety of XN. Furthermore, the potential use of XN as a food additive considering its many positive biological effects is discussed.

## 1. Introduction

**Figure 1:**
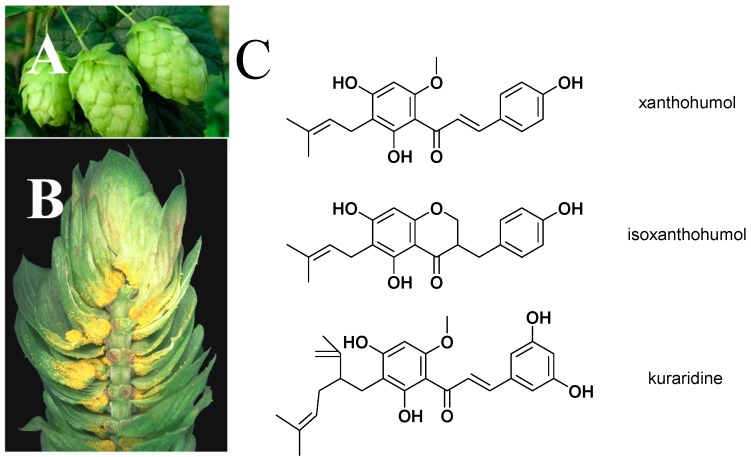
Female hop flowers (**A**); hop flowers resin covering flower bracts (**B**); and structure of xanthohumol, isoxanthohumol, and kuraridine (**C**).

Hops (*Humulus lupulus* L.) flowers (Figure 1A,B) are widely used throughout the world as a raw material in the brewing industry, to preserve beer and to give beer its characteristic aroma and flavor. In addition to the application in the brewing industry, hops have for a long time been used for various medical purposes [1]. Prenylated flavonoids are one kind of bioactive substances contributing to its medical applications. The most abundant prenylated flavonoid in hops is xanthohumol (XN, Figure 1C). In nature, XN exists ubiquitously within hops plant, with a content of 0.1%–1% (dry weight) in the female inflorescences. XN is secreted mainly as part of the hop resin and is also found in the trichomes on the underside of young leaves. The conventional XN isolation method was to use repeated chromatographic steps on silica gel using different solvents [2], and the recently established efficient way for the isolation and purification of XN from hops extract is by means of a high-speed counter-current chromatography method [3]. A chemical synthesis method to synthesize XN using phloracetophenone (2',4',6'-trihydroxyacetophenone) as precursor has been established. However, the process is complicated and the overall yield is relatively low [4]. Thus, extraction, isolation, and purification from female inflorescences is still the main method to obtain XN.

Beer is the most important dietary source of XN and related prenylflavonoids. However, XN is generally a minor prenylflavonoid in beer due to the thermal isomerization of chalcones into flavanones, in this case isoxanthohumol (IX), occurring during the brewing process [5]. In commercial beers, less than 0.2 mg·XN/L is found, which is not enough to really be beneficial to the health. In order to increase the yield of XN in the brewing process, the parameters of XN recovery were modified, including the use of XN-enriched hop products, the use of special malts, the late addition of hops to the boiling worts, and control of the temperature for addition of XN products to sweet worts [5,6,7]. Consequently, brewing technology that produces beer with high XN content has been established on an industrial scale [6,7]. The daily intake of XN is relatively small compared to total polyphenols from beer [8], indicating that XN contributes little to the antioxidant properties of beer. However, XN is more lipophilic and therefore possibly possess more bioactivity than other beer polyphenols [9].

The structure of XN was first identified by Verzele in 1957 [10], but only in the last decade, was XN rediscovered, focusing in particular on its multiple bioactivities, including anticancer, antidiabetic, antibacterial, anti-inflammatory activities, and so on. The pharmacological activity of XN has gained much attention in the functional food and pharmaceutical area. In this review, we focus on the bioactivities, pharmacokinetics, safety, and potential application of XN in pharmaceutics, since there are only a few reviews related to the possible benefit of XN to human beings [11,12].

## 2. Bioactivity, Pharmacokinetics, and Safety of XN

### 2.1. Effect on Metabolic Syndrome and Related Disorders

Metabolic syndrome is a group of risk factors that include hyperglycemia, abdominal fat, disordered cholesterol levels, and high blood pressure. Scientifically validated plant-based interventions are a practical means of addressing the epidemics of the metabolic syndrome [13]. Experimental research revealed that XN can attenuate several factors of the metabolic syndrome as described below.

#### 2.1.1. Anti-Obesity Activities

XN has been reported to inhibit adipogenesis or increase cell apoptosis and therefore can be used in preventing obesity. In 3T3-L1 cells, both the purified XN and hops extract rich in XN inhibit the differentiation of preadipocytes by decreasing the major adipocyte marker proteins such as peroxisome proliferator-activated receptor (PPAR) γ, CCAAT enhancer binding proteins (C/EBP) α, and fatty acid binding protein (aP2) [14,15,16]. In addition, XN also induces apoptosis in mature adipocytes through the mitochondrial pathway [14,15]. The differention and apoptosis activity of XN on adipocytes are enhanced when used combined with guggulsterone and honokiol [17,18]. As well as effecting adipocytes, XN also effects the bioenergetics of muscle cells. XN may attenuate the metabolic syndrome, at least in part, through mitochondrial uncoupling and stress response induction [19]. Recent research reported that, feeding rats high-fat diet enriched with hop extract, XN inhibits the increase of body weight, liver weight, and triacylglycerol level in the plasma and the liver. The mechanisms are related to the regulation of the hepatic fatty acid metabolism and inhibition of fat absorption in the intestine [20]. It is interesting that α-mangostin with a structure akin to XN is shown to inhibit intracellular fatty acid synthase [21]. XN exerts preventive function on the increase of body weight induced by overnutrition, however, further clinical investigations are needed to confirm this effect, and the molecular mechanisms for this effect have yet to be found.

#### 2.1.2. Hypoglycemic Activities

Nutritional approaches using phytonutrients for the prevention or treatment of type 2 diabetes mellitus (T2DM) are a rapidly emerging trend. XN has been reported to enhance the metabolism of plasma glucose [19,22]. A high XN dose (16.9 mg/kg) exerted beneficial effects on body weight and glucose metabolism in obese male rats [22]. This suggests that XN holds promise as a therapeutic agent for treating obesity and dysregulation of both glucose metabolism and the metabolic syndrome [19,22]. Levels of plasma glucose, plasma, and hepatic triglyceride in KK-A^y^ mice decreased when fed with XN. The XN-fed mice also showed decreased amounts of water intake, lowered weights of white adipose tissue, and exhibited increased levels of plasma adiponectin. This investigation indicates that XN boosts glucose metabolism and attenuates diabetes in KK-A^y^ mice. The mechanisms are possibly related to XN acting as a ligand of the farnesoid X receptor, which is positively correlated with lipid accumulation, and regulates downstream gene expression [23]. Moreover, research showed consumption of XN by diabetic animals consistently decreases inflammation and oxidative stress, allowing neovascularization control and improving complicated diabetic wound healing [24]. The inhibition of glucose uptake in intestinal cells [25], as well as the inhibition against α-glucosidase [26] may also contribute to the hypoglycemic activity of XN. XN has a Michael acceptor moiety that can covalently interact with proteins. The inhibition against α-glucosidase is possibly attributed to a Michael-type addition of cysteine residues to the α,β-unsaturated keto group of XN [26]. Since the Michael reactions are reversible, release of XN recovers the enzyme function in a dialysis experiment and thus explains the reversible inhibitory mode [26]. In addition, IX (see Figure 1), a spontaneous cyclization product of XN with no electrophilic properties and no ability for Michael addition, does not possess an obvious inhibition effect against α-glucosidase [27]. On the other hand, kuraridine, which has a skeleton similar to XN and contains the α,β-unsaturated keto group, possesses much stronger inhibitory activities against α-glucosidase [27]. Therefore, α-glucosidase is one of the possible targets of XN. All of these findings strongly indicate that XN has potential benefits in the treatment of obesity and diabetes.

#### 2.1.3. Anti-Hyperlipidemia Activities

In the HepG2 cell model, XN inhibits the synthesis of triglyceride (TG) in the microsomal membrane and the transfer of the newly synthesized TG to the microsomal lumen [28]. Moreover, XN decreases apolipoprotein B (ApoB) secretion in a dose-dependent manner under both basal and lipid-rich conditions and this decrease is associated with increased cellular ApoB degradation. These results indicate its potential use in the treatment of hypertriglyceridemia [28]. Research also showed that XN is a diacylglycerol acyltransferase inhibitor [29,30,31], which is involved in triglyceride synthesis.

High density lipoprotein (HDL)-cholesterol levels are correlated with a low risk of atherosclerosis [32]. The inhibition of cholesteryl ester transfer protein (CETP), which catalyses cholesterol transfer between lipoproteins, leads to an increase in HDL-cholesterol. CETP is expected to be the next anti-atherogenic target. XN has been reported to possess potent inhibition against CETP in a mixed non-competitive inhibition mode, and the structure-activity-relationship study showed that the chalcone structure and prenyl group is necessary for its inhibitory activity [33]. The inhibitory potency of XN against endogenous CETP activity was confirmed *in vivo*. Via CETP inhibition and the apolipoprotein E (ApoE) enhancement, XN prevents cholesterol accumulation in atherogenic regions by HDL-cholesterol metabolism in CETP-transgenic mice fed with XN *ad libitum* for 18 weeks [34]. In Western-type diet-fed ApoE-deficient (ApoE^−/−^) mice, XN also ameliorates atherosclerotic plaque formation [35]. The mechanisms are related to their positive effect on plasma cholesterol levels, monocyte chemo attractant protein-1 (MCP-1) concentrations, and hepatic lipid metabolism via activation of AMP-activated protein kinase (AMPK) [35].

Oxidation of low-density lipoprotein (LDL) is thought to play a central role in atherosclerosis [36]. Being a chalcone, XN also possesses superoxide scavenging capacity [37]. XN shows high antioxidant activity in inhibiting LDL oxidation. When combined with α-tocopherol, XN completely inhibits copper-mediated LDL oxidation. According to these findings, XN protects human LDL from oxidation [38]. XN modulates the lipid metabolism and therefore prevents cardiovascular diseases such as atherosclerosis. Besides its direct antioxidant activity, XN also induces cellular defense mechanisms to overcome the oxidation stress induced by chemicals [39] or surgery [40].

### 2.2. Cancer Related Bioactivities

Cancer is an abnormal and uncontrollable multiplication of cells or tissue. Agents that inhibit the initiation, promotion, and progression stages of carcinogenesis, consist of a broad spectrum of chemo-preventive candidates for cancer treatment. In recent years, experimental results of a number of studies have showed that XN can prevent and treat cancers [41]. The mechanisms of anticancer activity have been identified, including chemopreventive activity by inhibition of the initiation and development of carcinogenesis, and therapeutic activity by inhibition of proliferation, induction of apoptosis, and inhibition of migration and angiogenesis.

#### 2.2.1. Cancer Chemo-Preventive Effect

XN shows anti mutagenic activity against mutations induced by the food borne mutagen 2-amino-3-methylimidazo[4,5-f]quinoline (IQ) [42,43]. Using the *Salmonella*/microsomal assay system and human hepatoma HepG2 cells, XN prevents IQ induced DNA damage [42]. The mechanisms are possibly related to the inhibition of the metabolic activation of IQ by human cytochrome P450 1A2 (CYP1A2) and the binding of IQ metabolites to DNA and proteins [43]. Besides the protection against IQ induced genotoxicity, XN also protects DNA against benzo(a)pyrene (BaP)-induced oxidative stress and DNA damage in HepG2 cells [39], and in fresh liver tissue [44]. In HepG2 cells, XN results in significantly reduced *tert*-butyl hydroperoxide (an inducer of reactive oxygen species)-induced DNA strand breaks, indicating that its protective effect is mediated by induction of cellular defense mechanisms against oxidative stress [39]. Another study revealed that XN significantly reduces menadione induced DNA single-strand breaks in Hepa1c1c7 cell and shows good chemo-preventive activity through induction of quinone reductase [45,46]. The mechanism by which XN induces quinone reductase is through alkylation on kelch-like ECH-associated protein 1 (Keap1). Keap1 sequesters nuclear factor E2-related factor 2 (Nrf2) in the cytoplasm, which regulates the expression of the quinone reductase [46]. The anti-carcinogenic properties at the initiation, promotion, and progression stage of carcinogenesis have been investigated and the results showed that XN is a potent chemo-preventive agent [47]. Although the mechanism of the protective effect of XN is not yet fully elucidated, the accumulated results indicate that XN exhibits anti-genotoxic effects against many mutagens and provide evidence for its cancer preventative potential.

#### 2.2.2. Anti-Angiogenic Activity

New vascularization is necessary for tumor growth and metastatic dissemination. Thus, the inhibition of tumor angiogenesis is a promising strategy in cancer therapy and prevention. One of the main mechanisms of its anticancer activity, XN targets the endothelial and vascular cells, and shows inhibitory activities in tumor angiogenesis [48,49,50,51].

XN administrated to mice in their drinking water inhibits the growth of a vascular tumor *in vivo* via tumor angiogenesis inhibition [48]. Subcutaneous application of XN (l mg/g body weight) for 14 days to SCID mice bearing human MX-1 breast tumor xenografts significantly reduces the tumor-induced neovascularization by 30% [50]. The mechanisms for its inhibition of angiogenesis are related to the blockage of both the nuclear factor-κB (NFκB) and Akt pathways in endothelial cells [48]. XN interferes with several points in the angiogenic process, including inhibition of endothelial cell invasion and migration, growth, and formation of tubular-like structures in HUVEC cells and HMEC-1 cells [48,50]. The identical activity was observed also in human fetal aortic smooth muscle cells [49,51]. However, XN exhibits the opposite effect when HUVEC were co-cultured with human fetal aortic smooth muscle cells, leading to an increase in the number of cord structures, and showing no inhibitory effects in mature vasculature, indicating that XN mainly target the angiogenic, but not the stable vessels [49]. Moreover, besides the direct effect on the vascular cells, XN inhibits the production of angiogenic factors in pancreatic carcinoma cells and blocks the pancreatic cancer associated angiogenesis, e.g., vascular endothelial growth factor (VEGF) and interleukin 8 (IL-8). The inhibition of the angiogenic factors production is considered to be via the inhibition of NFκB [52].

In addition to the potential use in tumor angiogenesis, the potent anti-angiogenic activity of XN indicates that XN may be useful for the treatment of other angiogenesis-related diseases, such as endometriosis [53], and wound healing [24,54].

#### 2.2.3. Proapoptosis Activity and Modulation of Autophagy

Generally, both apoptosis and autophagy are tumor suppressor pathways. Apoptosis prevents the survival of cancer cells, while autophagy facilitates the degradation of oncogenic molecules, and therefore prevents the development of cancers. However, under stress conditions, autophagy also facilitates the survival of tumor cells [55]. Consequently, drug-induced apoptosis or modulation of autophagy can be effective strategies for treatment of cancer.

Many researchers have shown that XN exerts anticancer activities by inhibiting proliferation and inducing apoptosis of cancer cells. XN induces apoptosis of multiple kinds of cancer cells, including human prostate cancer [56,57], leukemia [58,59], ovarian cancer [60], hepatocellular carcinoma [61,62], breast carcinoma [63] and human malignant glioblastoma [64,65]. For example, XN showed strong anticancer activity against breast cancer MCF-7 and prostate cancer HT-29 cell lines and the inhibitions are stronger than the positive control cisplatin [66]. The flavonoid skeleton type and modification of the prenyl group may affect the anticancer activity, but it differs in different cell lines [66], indicating that multiple mechanisms or targets are involved. The XN induced apoptosis is mainly related to the up regulation of anti-apoptotic proteins [67], down regulation of pro-apoptotic proteins, and activation of procaspases [68], and it seems that both the death receptor and mitochondrial apoptosis pathway are activated by XN [69]. Oxidative stress response [64,70,71] and endoplasmic reticulum stress response [72] are also reported to be involved in the XN induced apoptosis. Additionally, the phosphorylation of extracellular-signal-regulated kinase 1/2 (ERK1/2) and rapidly accelerated fibrosarcoma-1 (Raf-1) pathway can also be activated by XN in medullary thyroid cancer cells [73]. XN has been shown to induce apoptosis by inhibiting NFκB activation [74]. Other mechanisms are also involved in the XN induced apoptosis. For example, inhibition of topoisomerase I [75] and aromatase activities [76], as well as the decrease in the production of nitric oxide [77].

XN may also be a potent chemo- and radio-therapy sensitizer leading to apoptosis. XN sensitizes MCF-7/ADR cells to radiation treatment [78]. When treated with XN, multi-drug resistance 1 (MDR1), epidermal growth factor receptor (EGFR), and signal transducer and activator of transcription 3 (STAT3) decreases in MCF-7/ADR cells, while the death receptor (DR)-4 and DR-5 expression increases [78]. XN markedly augments the anticancer activity of tumor necrosis factor related apoptosis-inducing ligand (TRAIL) and sensitize TRAIL-resistant cancer cells by engaging extrinsic apoptotic pathway, with increased expression of DR-5 receptor in HeLa cells [79], and LNCaP prostate cancer cells [80]. XN, together with IX, 6PN, and 8PN, is inhibitor of the efflux transporter breast cancer resistance protein (BCRP/ABCG2), indicating its importance for xenobiotic bioavailability and multidrug resistance [81]. However, to the best of our knowledge, there is no report about the *in vivo* inhibition and delay of tumor growth.

In addition to the potential therapy on solid tumors, XN has an obvious inhibitory effect on the non-solid tumors, such as leukemia [82]. XN kills B-chronic lymphocytic leukemia cells by apoptosis [82]. XN induces apoptosis in K562 chronic myeloid cells via elevation of intracellular reactive oxygen species (ROS). XN inhibits Bcr-Abl expression at both mRNA and protein levels [58]. Furthermore, XN induced apoptosis in leukemic cells is related to the inhibition of NFκB, via modification of cysteine residues of the IκBα kinase and NFκB by XN [59]. Administration of 50 mg·XN/mouse (5 days/week) significantly increased animal life span by delaying the insurgence of neurological disorders due to leukemic cell dissemination [83]. Therefore, XN represents a promising agent for leukemia therapy, although clinical testing is needed in the near future.

Autophagy is a bulk, nonspecific protein degradation pathway that is involved in the pathogenesis of cancer and neurodegenerative disease. Recent research indicated that XN impairs autophagosome maturation of human epidermoid carcinoma A431 cells. The mechanism involves XN binding directly to the *N*-domain of valosin-containing protein (VCP), and acts as a VCP inhibitor. VCP is an essential protein for autophagosome maturation [84]. The modulation of autophagy by XN possibly contributes to the mechanisms underlying the anticancer activity of XN, although further studies are needed to illustrate whether this autophagy inhibit or facilitate the XN induced apoptosis.

#### 2.2.4. Anti-Invasion Activities

Metastasis, a characteristic of highly malignant cancers with poor clinical success has been one of the major causes for the increased mortality rate in cancer patients. Therefore, the inhibition of cancer cell invasion is very important for effective therapies against cancer.

XN is able to inhibit the invasion of human breast carcinoma MCF-7/6 cells in the chick heart invasion assay and of T47-D cells in the collagen invasion assay [85]. The mechanism of the anti-invasive effect of XN is related to the up regulation of E-cadherin/catenin invasion suppressor complex [85]. Prostaglandin E2 (PGE2) actuates several pathways implicated in chronic inflammation-related cancer. XN has potential to suppress the migration ability of cholangiocarcinoma cell lines by inhibiting PGE2 production [86]. Matrix metalloproteinases (MMPs) have been strongly implicated in multiple stages of cancer progression, including the acquisition of invasive and metastatic properties. XN shows strong inhibition on the invasive phenotype in estrogen receptor, progesterone receptor and human epidermal growth factor receptor 2 negative breast cancers cells, via down regulation of MMP-2 and/or MMP-9 [67]. Cysteine X Cysteine (CXC) chemokine receptor 4 (CXCR4) is overexpressed in various tumors and mediates homing of tumor cells to distant sites expressing its cognate ligand, CXCL12. XN suppresses CXCR4 expression in cancer cells at the transcriptional level via blocking endogenous activation of NFκB, which regulates the expression of CXCR4 in cancer cells. Consequently, XN abolishes cell invasion induced by CXCL12 in both breast and colon cancer cells [87]. Moreover, XN inhibits circular chemorepellent-induced defect formation in lymphendothelial cell monolayers, by inhibiting the activity of cytochrome P450, selectin E, NFκB, and the expression of intercellular adhesion molecule 1 (ICAM-1) [88]. XN decreases the adhesion of tumor cells to endothelial cells, via inhibiting the markers of epithelial-to-mesenchymal transition and of cell mobility such as paxillin, myosin light chain 2, and S100A4 in breast cancer cells [88]. XN also inhibits leukemia cell invasion, metalloprotease production, and adhesion to endothelial cells [58], and therefore also has potential to preventing *in vivo* life-threatening complications of leukostasis and tissue infiltration by leukemic cells. The potential activity against both migration and invasion indicates a possible role of XN as an anti-invasive agent *in vivo* as well.

### 2.3. Anti-Inflammatory Activity

Nitric oxide (NO) plays an important role in many inflammatory responses and is also involved in carcinogenesis. In mouse macrophage RAW264.7 cells, XN (10 μg/mL) inhibits more than 90% of the NO production by suppressing inducible NO synthase (iNOS) induced by a combination of lipopolysaccharide (LPS) and interferon-γ (IFN-γ) [77].

Further studies on the anti-inflammatory activity of XN showed that different signaling pathways are involved in macrophages. For example, when treated with LPS, XN reduces the expression of the LPS receptor components such as Toll-like receptor-4 (TLR4) and myeloid differentiation protein 2 (MD2) and results in the suppression of NFκB activation [89,90]; while in the IFN-γ stimulated RAW264.7 cells, XN inhibits the binding activity of STAT-1α and interferon regulatory factor-1 [89].

Excess levels of IL-12 in immune responses such as inflammation or autoimmunity have raised considerable interest in IL-12 blocking agents. XN inhibits IL-12 production in stimulated macrophages through the down regulation of NFκB [91]. The *in vivo* anti-inflammatory effect of XN using an oxazolone-induced chronic dermatitis model in mouse ear was evaluated, and the results showed that dermatitis is attenuated by XN, indicating the potential application of XN in the treatment of skin inflammation [91].

Cytokine IL-2 plays an important role in the acquired immune responses via T cells. In phorbol 12-myristate 13-acetate (PMA) and ionomycin activated EL-4 T cells, XN treatment induces a significant increase of the IL-2 production at the transcriptional level. Enhanced activity of the IL-2 promoter, and the up regulation of several transcription factors modulating of IL-2 expression, such as nuclear factor of activated T cells (NF-AT) and activator protein-1(AP-1), contributes to the increase of the IL-2 production [92]. Another study showed that XN has profound immunosuppressive effects via modulating the T cell mediated response [93]. This suppression of T cell-mediated immune responses by XN includes T cell proliferation, development of IL-2 activated killer cells, cytotoxic T lymphocytes, and production of Th1 cytokines (IL-2, IFN-γ, and TNF-α). The immunosuppressive effects are possibly due to the inhibition of NFκB through suppression of phosphorylation of IκBα [93].

XN also reduces the release of several inflammatory factors, such as monocyte chemo attractant protein-1 (which plays a crucial role in the inflammatory response) and tumor necrosis factor-γ (TNF-γ) in LPS-stimulated RAW 264.7 mouse macrophages and U937 human monocytes [94]. Besides XN, other plant-derived polyphenols, e.g., mangostin and kaempferol, can also down regulate TNF and other proinflammatory biomarkers [95].

XN inhibits LPS-stimulated inflammatory responses in microglial BV2 cells via the Nrf2 pathway and upregulates the antioxidant enzymes, NQO1 and HO-1. XN regulates Nrf2 signaling and indicates its potential use in the prevention of neurodegenerative diseases associated with inflammation [96].

In addition to the regulation of inflammatory factors, XN also shows direct effect on immune cells. Dendritic cells (DCs) are key players in the regulation of innate and adaptive immunity. XN induces apoptosis of bone marrow-derived DCs via acid sphingomyelinase stimulation and caspase activation [97].

The multiple targets and mechanisms of XN may explain its broad anti-inflammatory effects. The broad spectrum of anti-inflammatory activity *in vitro* indicates its potential in treating various diseases associated with inflammation. As inflammation has a close relationship with cancer, it is speculated that its anti-inflammatory activity may be also one of the possible mechanisms for its anticancer activity. However, most of the studies so far are at the *in vitro* level, and more *in vivo* studies are needed to confirm the anti-inflammatory efficacy.

### 2.4. Central Nervous Systems Modulation Properties

β-Site amyloid precursor protein (APP) cleaving enzyme 1 (BACE1) mediates cleavage of β-APP and facilitates learning, memory, and synaptic plasticity. It has been proven that BACE1 is a potential target for Alzheimer’s disease. BACE1 activities are significantly inhibited by XN with an IC_50_ value of 7.19 μM [98]. Therefore, XN may be a potent preventive and therapeutic candidate for Alzheimer’s disease [98]. XN exerts neuro protective effects on cerebral ischemic damage in rats. XN results in reduction of the infarct volume and the improvement of neuro behavior in cerebral ischemic rats. The mechanism is probably related to its inhibition of inflammatory responses (*i.e.*, increase of hypoxia-inducible factor-1α, (HIF-1α), iNOS expression, and free radical formation), apoptosis (*i.e.*, TNF-α, active caspase-3), and platelet activation [99], indicating its therapeutic potential for treatment or prevention of ischemia-reperfusion injury-related disorders. Further investigations showed that derivatives of XN can induce neurite growth in mouse neuronal cells [100]. XN has sedative effects due to binding to GABA_A_ receptors and hindering the lateral mobility in neurons [101], and this may explain why hops are traditionally useful in treating sleeplessness and nervousness. However, other compounds in hops can also be at play [1]. Furthermore, in an evaluation of the anxiolytic effects of XN using the Sprague-Dawley rat model, the results showed that modulation of the GABA_A_ receptor does not contribute to the anxiolysis produced by XN [102]. XN possibly influences other neurotransmitter sites in the central nervous system [102]. In the brain of female senescence accelerated mouse, XN improved pro-survival signals and reduces pro-death signals in age-related impairments of neural processes [103]. Dietary intake of XN was shown to improve cognitive flexibility in young mice and to lower plasma palmitate in young and old mice [104]. Generally, the higher level of protein palmitoylation is considered to be associated with poorer learning scores.

These combined results show that XN has beneficial effects in the central nervous system. However, the number of clinical studies supporting the use of XN as a central nervous systems modulator is rather limited and the effect of XN at the central nervous system requires a thorough reinvestigation.

### 2.5. Antimicrobial Activity

The discovery of novel antimicrobial agents has been going on for many years. However, the new drugs have not kept pace with the increasing drug resistance. One of the major challenges is the limitation of screening libraries. Natural plant products, such as chalcones, may contribute to the improvement of these chemical libraries.

XN inhibits human immunodeficiency virus (HIV-1) induced cytopathic effects, the production of viral p24 antigen and reverse transcriptase in C8166 lymphocytes [105]. XN also moderately inhibits HIV-1 replication in peripheral blood mononuclear cells with an EC_50_ value of 20.74 µg/mL, but does not inhibit the activity of recombinant HIV-1 reverse transcriptase and HIV-1 entry [105]. The results suggest that XN is effective against HIV-1 and may serve as an interesting lead compound for development of anti-HIV agents. Besides HIV, XN also inhibits the bovine viral diarrhea virus (BVDV), the herpes viruses (HSV-1, HSV-2 and CMV) with a low-to-moderate extent [106], and inhibits the hepatitis C virus (HCV) replication in cell culture systems, comparable to IFN-α [107].

Studies showed that XN displays a broad spectrum of anti-infective activities against bacteria such as *Staphylococcus aureus* [108] and *Streptococcus mutans* [109]. A recent study has shown that XN inhibits the growth of *Staphylococcus aureus* strains with a MIC range of 15.6–62.5 µg/mL and shows potent anti-adherent and anti-biofilm activity [110]. XN also shows anti-fungal activity as evidenced by the inhibition of two *Trichophyton* spp. [111].

The broad spectrum of antimicrobial activity of XN has been documented and reviewed [111], including the inhibition towards virus, bacteria, and fungi, but the detailed mechanisms of these antimicrobial inhibitory activities are still under investigation. Although it has a broad spectrum of antimicrobial activity, XN does not affect the composition of intestinal microbiota in rats [112], suggesting an unchanged profile for intestinal microbiota when XN is administrated *in vivo*.

### 2.6. Anti-Parasite Effects

Anti-coccidial effects of XN have been reported and the results showed that XN can reduce the invasion by *Eimeria tenella* sporozoites (SZ) in Madin-Darby bovine kidney cells and reduce the invasion by *E. tenella* and *E. acervulina* SZ in a chick host. This inhibition is associated with the disruption of the apical ends of the SZ [113]. XN results in significantly reduced gross-lesion scores and normal chick-host weight gains compared with untreated SZ, indicating XN could be used as anti-coccidial feed additive [113]. Moreover, XN and its chalcone derivatives inhibit the *in vitro* replication of *Plasmodium falciparum*, the major parasite causing malaria. The anti-plasmodial mechanisms may be related to interference with the glutathione-dependent haemin-degradation process of *P. falciparum* [114].

### 2.7. Effect on Bone Disease

Bone remodeling is a dynamic process which is maintained by a balance between bone formation and bone resorption. XN has a strong inhibitory effect on bone resorption, and is speculated as a precursor of phytoestrogen compounds because the demethylxanthohumol is a proestrogen and is metabolized to the active estrogenic compound prenylnaringenin *in vivo* [115]. XN dose-dependently stimulates osteogenic marker gene (Runx2, ALPL, and BGLAP) expression as well as ALPL activity in murine mesenchymal and pre-osteoblast cell lines, reciprocally affecting the osteogenic *versus* the adipogenic differentiation pathway [116]. However, XN does not show progestogenic or androgenic bioactivity, and the endocrine properties of hops and hop products are due to the estrogenic activity of 8PN [117]. Receptor activator NFκB ligand (RANKL) has been shown to play a critical role in osteoclast formation and bone resorption. The newest research showed that XN markedly inhibits RANKL-induced tartrate-resistant acid phosphatase activity, multinucleated osteoclasts formation, resorption-pit formation, and modulates the expression of osteoclast-specific genes during osteoclastogenesis in RAW264.7 cells [118]. These results indicate that XN inhibits osteoclastogenesis and may be useful for the prevention of bone diseases.

An early reaction in osteoarthritic chondrocytes is hyaluronan overproduction followed by proteoglycan loss and collagen degradation. XN inhibits hyaluronan export [119,120], as well as proteoglycan and collagen loss, and prevents the shedding of metalloproteases into the culture medium [119]. The mechanism is that XN directly binds and inhibits the hyaluronan exporting protein, multidrug resistance associated protein 5 (MRP5) [119,121], while not influencing the hyaluronan synthase activity [119]. Therefore, XN may be a natural compound to prevent hyaluronan overproduction and subsequent reactions in osteoarthritis.

### 2.8. Hepatic Protection

#### 2.8.1. Protection in Chemical Hepatic Injury

XN has the potential as functional nutrient for prevention or treatment of non-alcoholic steatohepatitis. Hepatocytes and hepatic stellate cells (HSC) are central mediators of liver fibrogenesis. XN inhibits the activation of primary human HSC and induces apoptosis in activated HSC *in vitro* without impairing viability of primary human hepatocytes. XN inhibits the activation of NFκB and the expression of NFκB dependent pro-inflammatory genes [122]. *In vivo*, feeding of XN reduces hepatic inflammation and inhibits the expression of profibrogenic genes in a murine model of non-alcoholic steatohepatitis [122]. In addition, in a liver injury rat model induced by carbon tetrachloride (CCl_4_) [123,124] and in a hepatocyte model induced by *tert*-butyl hydroperoxide (TBH) [125], XN shows obvious protective effects against toxic liver injury. The mechanisms are related to the inhibition of hepatic inflammation via decreasing NFκB activity [123], inhibition of lipid peroxidation [124,125], and protection against the degradation of antioxidant enzymes [124]. XN induces the detoxification enzyme, NAD(P)H-quinone oxidoreductase (NQO1) *in vitro* and in the liver [126], by modifying Keap1, which induces Nrf2 translocation and antioxidant response element activation [126]. The mechanisms are similar to that in the cancer chemo-preventive and anti-inflammation effect, as are result of Keap1 alkylation and the resulting activation of antioxidant enzymes are also involved [46,96]. XN acts as a protective agent against oxidative damage induced in rat liver and other tissues after acute intoxication due to ethanol administration [127]. In normal hepatocytes, the chemopreventive activity of XN may relate to the activation of Nrf2, phase II enzymes, and induction of p53 [61]. These studies further indicate the potential application treatment of liver fibrosis in response to hepatic injury.

#### 2.8.2. Protection in Liver Ischemia/Reperfusion Injur

Liver ischemia/reperfusion (I/R) leads to the formation of ROS, causing hepatic injury and initiating an inflammatory response, which is a critical problem after liver surgery and transplantation. In a mouse model of warm I/R liver injury, I/R-induced oxidative stress was significantly inhibited by XN. The mechanism is related to the inhibition of AKT, NFκB, and the proinflammatory genes [40]. However, in a cold I/R model, XN does not protect against I/R injury in rat liver [128]. The reason for the conflicting observations is possibly due to the different models, experimental conditions, and the XN concentration in the experiments [40].

#### 2.8.3. Benefits in Liver Diseases Associated with Virus Infection

HCV infection is a one of the major causes of liver infectious diseases. *In vitro* studies using BVDV, a model of HCV, showed that XN inhibits BVDV replication and enhanced the anti-viral activity of IFN-α [129,130]. In *in vivo* HCV infected *Tupaias*, XN reduces hepatic inflammation, steatosis, and fibrosis. The mechanisms are related to the inhibition of oxidative reaction, regulation of apoptosis, modulation of microsomal triglyceride transfer protein activity, and inhibition of hematopoietic stem cells [131].

### 2.9. Effects on Skin Disease

A study of XN on melanogenesis using B16 melanoma cells showed that XN might act as a hypo-pigmenting agent through the down regulation of microphthalmia-associated transcription factor (MITF) in the cAMP-dependent melanogenic pathway [132]. XN inhibits against collagenase activities (MMP-1 and MMP-8) and attenuates the oxidative damage to the skin, which are beneficial to the pathogenesis of acne vulgaris [108]. XN improves skin structure and firmness, mainly through inhibition of the elastase activity and MMPs and stimulating the biosynthesis of fibrillar collagens, elastin, and fibrillins [133]. Therefore, XN has potential as an anti-skin-aging agent. More physiological effects on skin health of XN and other beer compounds have been reviewed recently [134]. Potential uses of these substances in dermatology may include treatment of atopic eczema, contact dermatitis, pigment disorders, skin infections, skin ageing, skin cancers, and photo protection.

### 2.10. Thyroid Diseases

Sodium-iodide-symporter, an integral plasma membrane glycoprotein, mediates the sodium-dependent active uptake of iodide into the thyroid gland, which is a fundamental step in thyroid hormone synthesis. Recent reults have shown that nanomolar concentrations of XN stimulates the uptake of iodide in rat thyrocyte cells. Therefore, XN may be an interesting candidate for more efficient radioiodide therapy of the thyroid [135]. In additon, XN has an effect on certain drug transporters and modulates the transport of several drugs [136,137,138]. XN also affects the thyroid hormone distribution and metabolism by modulation hepatic expression of sulfotransferase, uridine-diphosphate glucuronosyltransferase, and the constitutive androstane receptor [139].

### 2.11. Benefits in the Thromboembolic Disease

Blood platelet activation and aggregation contributes to the atherothrombotic events. Studies showed that XN possesses potent antiplatelet activity via inhibition of the PI3-kinase/Akt, p38 MAPK, and PLCγ2-PKC pathways, and the inhibition on thromboxane A2 formation and [Ca^2+^]_i_ [140]. XN inhibits suicidal erythrocyte death induced by oxidative stress and energy depletion *in vitro*. Since eryptotic cells are cleared from the circulating blood and impede microcirculation, this novel effect of XN may be used in the prevention or treatment of anemia and disorders of microcirculation and coagulation [141]. Therefore, XN has potential in the treatment of thromboembolic disorders.

### 2.12. Pharmacokinetics and Biotransformation of XN

**Scheme 1:**
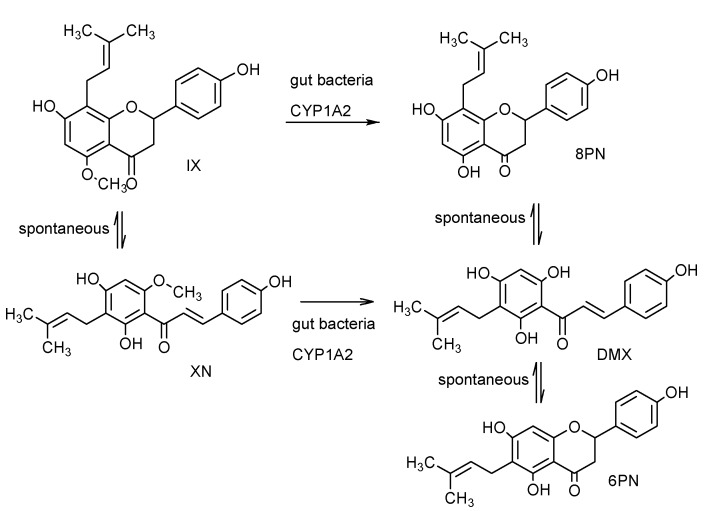
Pathway for XN metabolism and production of its metabolites: IX, 6PN, 8PN, and DMX. Reproduced from reference [152].

In rat and human liver microsomes, XN can be biotransformed to glucuronides and hydroxylated metabolies and cyclic dehydro-metabolites [142,143]. Investigations using human liver microsomes showed that hydroxylation of a prenyl methyl group is the primary route of the oxidative metabolism, forming hydroxylated metabolites of XN and IX. IX may be *O*-demethylated by human hepatic cytochromes P450 or gut microbial enzymes to form 8PN (Scheme 1). An important possibility is that XN may be converted into IX in the stomach which again may be converted to 8PN. Some of the effects of XN may therefore in effect be caused by 8PN [144,145]. XN can also undergo direct metabolic conversion to desmethylxanthohumol (DMX), which is later converted into either 6-prenylnaringenin (6PN) or into 8PN [146]. Both 6PN and 8PN are strong phytoestrogens. Studies in menopausal women to evaluate safety and pharmacokinetics of extract of hops also confirmed demethylation of IX to form 8PN and cyclization of XN to IX [147].When XN is fed to rats in a dose of 1000 mg·kg^−1^ body weight, feces is the major route of excretion [148,149]. 22 metabolites are identified in the feces, most of them confined to modified chalcone structures and flavanone derivatives [150]. However, indicating most of the XN remains unchanged in the intestinal tract of as approximately 89% is XN and only 11% is metabolites [150]. Phase II metabolites of XN in rats are also identified revealing oxidation, demethylation, hydration and sulfatation reactions [151]. Due to the multiple biotransformation of XN, we should keep in mind that, some of the metabolites of XN may contribute to the biological activity of XN, such as the estrogenic activity of 8PN and 6PN, and the products of biotransformation of XN, together with other prenylated hop flavonoids, could serve as an inspiration for drug design [66].

The pharmacokinetics of XN both in rats and in humans have been studied and provide pharmacokinetics parameters for XN [146,152]. The bioavailability of XN is dose-dependent and approximately 0.33, 0.13, and 0.11 in rats, when single orally administrated 1.86, 5.64, and 16.9 mg/kg body weight [152]. Human pharmacokinetics results showed that, following oral administration, XN shows a linear response with increasing oral dose, and XN has a distinct biphasic absorption pattern. XN and IX conjugates are the major circulating metabolites [146]. Slow absorption after oral administration in human and enterohepatic recirculation contributes to long half-lives of XN [147]. The collected data from rats and human demonstrate that there is similarity in XN metabolism between animals and humans, allowing for translation of animal study findings to future clinical studies.

Due to its low bioavailability in the human organism, much work has been performed to investigate the actual concentrations and pharmacokinetics in liver and intestinal cells. XN can accumulate rapidly in intestinal cells and most of the XN molecules are bound to cellular proteins. About 70% of XN in the apical side of Caco-2 cells accumulates inside the cells, and 93% of the intracellular XN is localized in the cytosol, and facilitated transport is not involved in the uptake of XN [153,154]. This specific binding of XN to cytosolic proteins in intestinal epithelial cells may contribute to the poor oral bioavailability *in vivo* [154]. Studies have also been done to investigate the interaction of XN with phosphatidylcholine model membranes [155,156], using X-ray diffraction, Fourier transform infrared spectroscopy, differential scanning calorimetry, and fluorescence spectroscopy. The results showed that XN inserts into lipid bilayers and affects molecular organization and biophysical properties of the bilayer [155,156], and this interaction may contribute to the rapid transport through the cell membrane.

### 2.13. Safety of XN

Toxicological studies in animals revealed that XN possesses good tolerance. The oral administration of XN (5 × 10^−4^ M *ad libitum*) to mice for 4 weeks did not affect the major organ functions, nor the protein, lipid, and carbohydrate metabolism [157]. Similarly, female BALB/c mice fed on XN (1000 mg/kg body weight) for 3 weeks exhibit no adverse effects on major organ function and homoeostasis [158]. Another study reported subchronic 4-week toxicity as well as its influence on fertility and development of offspring [159]. Sprague Dawley rats were treated with 0.5% XN in the diet or with 1000 mg/kg body weight *per* day by gavage for 28 days. Weak hepatotoxicity and poor development of mammary glands are observed in rats [159]. Furthermore, administration with XN (100 mg/kg body weight *per* day), does not cause any adverse effects on female reproduction and the development of offspring. However, XN treatment of male rats prior to mating significantly increases the sex ratio of male to female offspring [159]. An escalating dose study was carried out in menopausal women to evaluate safety of hops extract rich in XN, and the results showed this extract does not affect the sex hormones or blood clotting and reveals no acute toxicity [147].

## 3. Conclusions

It is only recently that researchers showed an increasing interest in XN and especially its biological activities. The anti-inflammatory, antioxidant, hypoglycemic activities, anticancer effects, and so on, assessed both *in vitro* and *in vivo* studies, strongly suggest a potential prevention and treatment of many diseases. The antioxidant activity may contribute to several ROS related diseases by acting directly as reducing compound, or indirectly by inducing the cellular defense mechanisms to overcome the oxidant stress [38,39,40]. Some of the molecular targets for their bioactivity are identified, and the interaction between XN and the target has also been investigated, including alkylation on the cysteine residues of Keap1, IκBα kinase, and NFκB by Michael addition [46,59], as well as binding directly to the VCP [84] and MRP5 [121]. It should be noted that Keap 1 is an important target for the bioactivity of XN, such as cancer chemo-prevention, hepatic protection, anti-inflammation, because alkylation on Keap1 activates antioxidant enzymes, such as quinine reductase, NQO1, HO-1, via the regulations of Nrf2 [46,96,126]. *In vivo* and *in vitro* studies to assess their bioavailability, distribution, efficacy, and safety in animal models and on humans have been performed with promising results for humans. The most important dietary source of XN is beer. However, XN is a minor prenylflavonoid in beer due to thermal isomerization of chalcones into flavanones during the brewing process [5], and pharmacologically relevant concentrations cannot be reached by consumption of regular beer. Now, it is possible not only to isolate XN but also to stabilize it in liquids and foodstuff with a high concentration [160] despite its low solubility in water. Therefore, pharmacological relevant concentrations can be reached by oral administration of XN enriched functional food, e.g. XN enriched beer, tea, fruit juice, solid foods (such as menohop^®^ (Metagenics, San Clements, CA, USA). Furthermore, in relation to XN being used as a drug, there is still a lot of work to be done in order to develop XN as a reliable drug for specific therapeutic applications in the clinic.

## Abbreviation

6PN: 6-Prenylnaringenin
8PN: 8-Prenylnaringenin
ALPL: Alkaline Phosphatase
ApoB: Apolipoprotein B
BACE1: β-Site APP Cleaving Enzyme 1
BVDV: Bovine Viral Diarrhea Virus
CXC: Cysteine X Cysteine
CXCR4: Cysteine X Cysteine Chemokine Receptor 4
CYP: Cytochrome P450 Protein
DCs: Dendritic Cells
DMX: Desmethylxanthohumol
DR: Death Receptor
HCV: Hepatitis C Virus
HIV: Human Immunodeficiency Virus
ICAM-1: Intercellular Adhesion Molecule 1
IFN-γ: Interferon-γ
IL: Interleukin
iNOS: Inducible NO Synthase
IX: Isoxanthohumol
Keap1: Kelch-Like ECH-Associated Protein 1
LPS: Lipopolysaccharide
MD-2: Myeloid Differentiation Protein 2
MIC: Minimum Inhibitory Concentration
MMP: Matrix Metalloproteinase
MRP5: Multidrug Resistance Associated Protein 5
NFκB: Nuclear Factor κB
NO: Nitric Oxide
NQO1: NAD(P)H-Quinone Oxidoreductase
Nrf2: Nuclear Factor E2-Related Factor 2
PMA: Phorbol 12-Myristate 13-Acetate
Raf-1: Rapidly Accelerated Fibrosarcoma-1
RANKL: Receptor Activator NFκB Ligand
ROS: Reactive Oxygen Species
STAT: Signal Transducer And Activator Of Transcription
TLR4: Toll-Likereceptor-4
TNF-α: Tumor Necrosis Factor-α
VCP: Valosin-Containing Protein
XN: Xanthohumol
